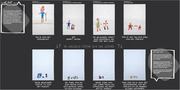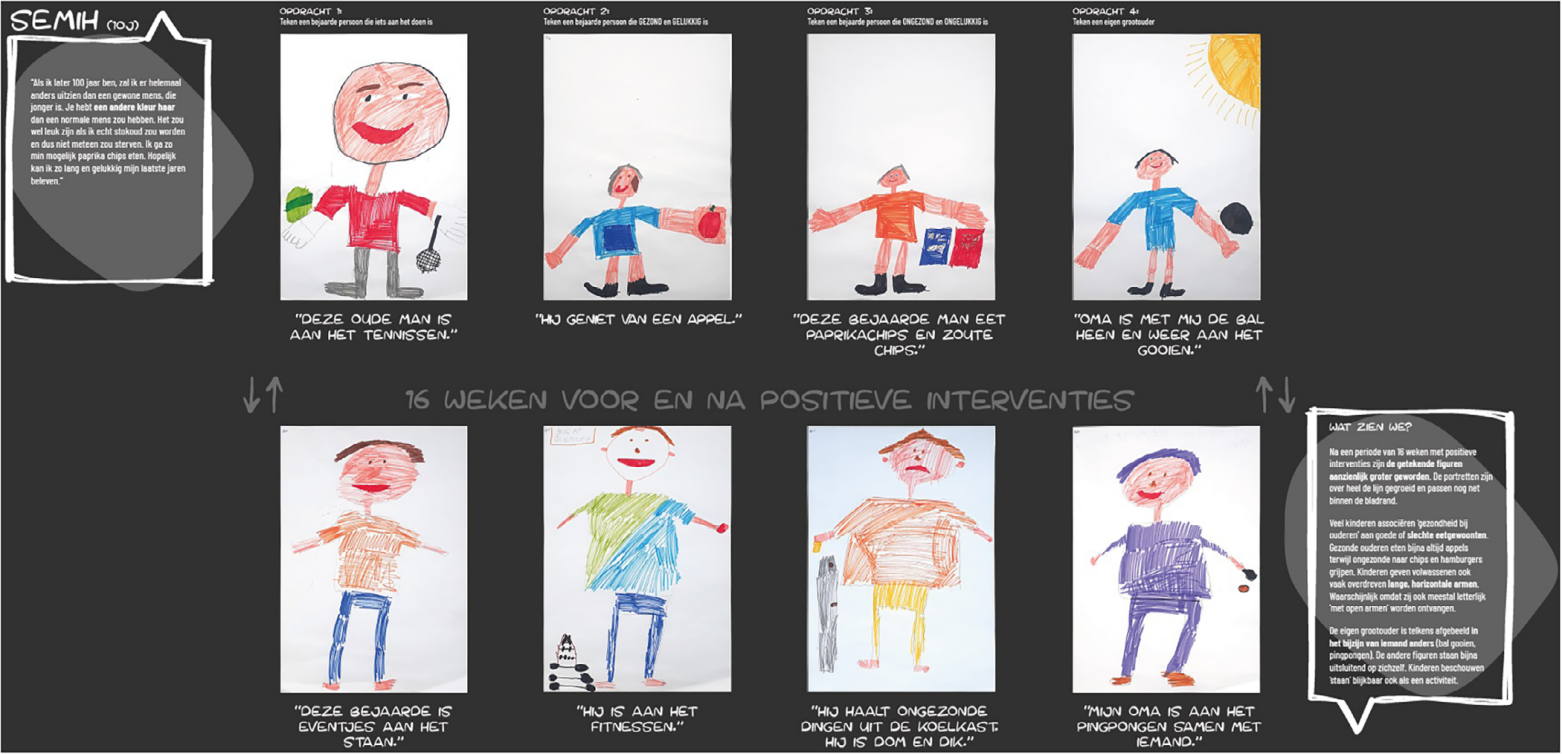# The Influence of COVID‐19 and Positive Interactions on Children’s Mental Representations of the Elderly and Dementia: A Drawing‐based Study

**DOI:** 10.1002/alz.086651

**Published:** 2025-01-09

**Authors:** Kasper Bormans, Sandra Zwakhalen

**Affiliations:** ^1^ Maastricht University, Maastricht Netherlands

## Abstract

**Background:**

Amidst the global impact of COVID‐19, this study delves into how restrictions and positive interactions shape children’s mental images of the elderly and dementia. We organized drawing sessions with three groups to investigate the malleability of children’s perceptions, offering crucial insights for future Alzheimer’s research.

**Method:**

This study gathered 848 children’s drawings from 106 ten‐year‐old children in Flanders and the Netherlands. Participants were asked to make four drawings: an older person, a healthy older person, an unhealthy older person, and their grandparent. The same drawing session is conducted before and after sixteen weeks. The children were divided into three groups: one group (N = 30) drew the elderly after a peak of COVID‐19 with strict restrictions, resulting in significantly smaller drawings (up to 33% on average). Another group (N = 34) drew the elderly after positive interactions with their grandparents and individuals with dementia, leading to considerably larger drawings (up to 21% on average), with more vibrant colours and more social elements. The third group (N = 42) drew the elderly without COVID‐19 restrictions and any specific intervention, resulting in a lack of changes in terms of size, colour usage, and content.

**Results:**

The findings suggest that the mental landscape of children is malleable. The different interventions, including exposure to COVID‐19 restrictions and positive interactions with elderly individuals, have a substantial impact on the size, colourfulness, and social aspects of the children’s drawings.

**Conclusion:**

This research provides important insights for future Alzheimer’s studies, particularly in the area of nuanced imagery surrounding ageing and dementia. The findings showed that children’s perceptions of the elderly may be influenced by various factors, highlighting the potential for interventions to shape their mental representations.